# DeepAnomaly: Combining Background Subtraction and Deep Learning for Detecting Obstacles and Anomalies in an Agricultural Field

**DOI:** 10.3390/s16111904

**Published:** 2016-11-11

**Authors:** Peter Christiansen, Lars N. Nielsen, Kim A. Steen, Rasmus N. Jørgensen, Henrik Karstoft

**Affiliations:** 1 Department of Engineering, Aarhus University, Aarhus 8200, Denmark; rnj@eng.au.dk (R.N.J.); hka@eng.au.dk (H.K.); 2 Danske Commodities, Aarhus 8000, Denmark; larsnn@gmail.com; 3 AgroIntelli, Aarhus 8200, Denmark; kas@agrointelli.com

**Keywords:** anomaly detection, obstacle detection, autonomous farming, precision agriculture, camera, background subtraction, change detection, DeepAnomaly

## Abstract

Convolutional neural network (CNN)-based systems are increasingly used in autonomous vehicles for detecting obstacles. CNN-based object detection and per-pixel classification (semantic segmentation) algorithms are trained for detecting and classifying a predefined set of object types. These algorithms have difficulties in detecting distant and heavily occluded objects and are, by definition, not capable of detecting unknown object types or unusual scenarios. The visual characteristics of an agriculture field is homogeneous, and obstacles, like people, animals and other obstacles, occur rarely and are of distinct appearance compared to the field. This paper introduces DeepAnomaly, an algorithm combining deep learning and anomaly detection to exploit the homogenous characteristics of a field to perform anomaly detection. We demonstrate DeepAnomaly as a fast state-of-the-art detector for obstacles that are distant, heavily occluded and unknown. DeepAnomaly is compared to state-of-the-art obstacle detectors including “Faster R-CNN: Towards Real-Time Object Detection with Region Proposal Networks” (RCNN). In a human detector test case, we demonstrate that DeepAnomaly detects humans at longer ranges (45–90 m) than RCNN. RCNN has a similar performance at a short range (0–30 m). However, DeepAnomaly has much fewer model parameters and (182 ms/25 ms =) a 7.28-times faster processing time per image. Unlike most CNN-based methods, the high accuracy, the low computation time and the low memory footprint make it suitable for a real-time system running on a embedded GPU (Graphics Processing Unit).

## 1. Introduction

Anomaly detection refers to the problem of finding patterns in data that do not conform to normal or expected behavior [1]. Using anomaly detection for obstacle detection will, instead of learning/classifying all object types or behavior, model the normal patterns and detects outliers. In an agricultural context, these outliers represent elements that are unnatural to the surrounding environment.

Conventional background subtraction (BS) algorithms are related to anomaly detection, as these methods subtract the background from the image, leaving behind only the foreground, which is an outlier to the background. Traditionally, BS methods model the background using color, intensity or gradients for each pixel using mixture of Gaussians [2], k-nearest neighbor or other classifiers to become invariant to small changes in illumination and moving shadows [3,4,5]. BS is intended for static cameras and for detecting moving or appearing objects in a video sequence. For a moving camera, the low level features used by conventional BS struggle to model a moving background [5], and many moving camera applications detect obstacles using general object detection algorithms or depth sensors.

Google Car (Google, San Jose, CA, USA) uses a Velodyne LiDAR as a depth sensor to perform convincing obstacle detection, and this is also a valuable sensor for obstacle detection in agriculture [6,7,8]. The drawbacks of using this sensor is the very high cost and that a depth sensor, especially in the automotive industry, exploits that an obstacle will protrude from the ground surface. In an agricultural context, obstacles may not protrude from the crop surface, introducing the risk of not detecting, e.g., kids, lying humans, hydrants, well covers and animals.

A camera-based system is much less expensive and, in principle, only requires obstacles to be visible and not necessarily protruding. In general, camera-based systems are less applicable for autonomous vehicles in terms of accuracy, range and computation time.

Mobileye is a company developing camera-based real-time systems for the automotive industry that are used in commercially available semi-autonomous vehicles, such as Tesla’s Model S. However, solutions by Mobileye are neither accessible to most researchers or trained for agriculture.

In research, deep learning perception algorithms and especially convolutional neural networks (CNN) [9,10,11,12,13] have improved the area of object detection [14,15,16,17,18] and semantic segmentation [19,20,21,22]. However, the training is performed on a predefined set of object types, and a large amount of annotated data is required for each object type.

To detect predefined object types, such as humans and some types of animals, data from various benchmarks [23,24,25] can be used in training. These benchmarks are not intended for agriculture and lack important object classes, such as tractors, fencing, shelter belts, water, etc. Most importantly, if such data were available, the algorithms would, by definition, not be able to detect other unspecified object types or unusual scenarios, e.g., a tent, a large red metal plate or a crashed road vehicle in the field. Secondly, in the context of agriculture, the object detection algorithm and semantic segmentation algorithms struggle to detect objects that are distant and heavily occluded by the crops.

In agriculture, the homogeneous characteristics of an agricultural production field and the fact that obstacles occur rarely and are of distinct appearance compared to the field should be exploited to detect non-predefined obstacles [26,27,28]. In [29], both distinct appearance (spacial analysis) and motion (temporal analysis) are used for detecting foreground elements.

**Figure 1:**
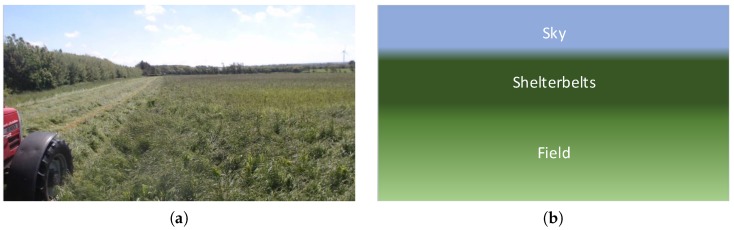
The visually homogenous characteristics of an agricultural field. (**a**) Shows agricultural field from tractor implement. (**b**) Illustration of the few visual components in an agricultural field.

In this work, the combination of background subtraction algorithms and high level features from a CNN is explored. Low level features used in conventional background subtraction algorithms are replaced with high abstraction features from a CNN. High abstraction features are less invariant to changes in pixel intensities caused by a moving camera and more dependent on actual image content. The intuition is that feature activations are nearly constant for grass, shelter belt or sky for a moving camera until completely new content is introduced in the image. A network trained for image classification on the ImageNet data [25] with 1000 different object types, targets the network features to activate especially on objects. The background model will more easily model the passive features of the background and detect feature activations from foreground objects. Secondly, the method exploits that images taken from a camera in motion (e.g., a tractor) have similar visual characteristics along image rows, as illustrated in Figure 1.

To our knowledge, limited research has combined deep learning with background subtraction or anomaly detection for obstacle detection in agriculture. In [30], a non-convolutional autoencoder has been used to dynamically reconstruct the background and detect foreground elements. In [31], the concept is similar to this work, as high level convolutional features are used in detecting foreground elements. The method is dependent on either human annotations or a simple background subtraction algorithm to initially generate training data. A critical drawback of both [30,31] for a tractor mounted camera is that they are developed for a static camera.

More deep learning research has been dedicated to an area related to anomaly detection called visual saliency [32]. The critical point of the visual-saliency is that it will always find a salient element no matter the image content. In agriculture, obstacles occur rarely, and an image is expected to mostly not contain an anomaly.

An elaborated investigation on combining background subtraction and deep learning is performed. We define a top performing configuration as DeepAnomaly, an anomaly detector that exploits the homogenous characteristics of an agricultural field. As an object detector, it is fast and has high accuracy, compared to the state-of-the-art. DeepAnomaly is intended to assist and not replace CNN-based object detection and semantic segmentation algorithms, when objects are distant, very occluded or unknown. Another property when used in conjunction with other CNN-based methods is that DeepAnomaly will only add little computational cost, as it may use features from another CNN-based obstacle detector.

## 2. Materials

**Figure 2:**
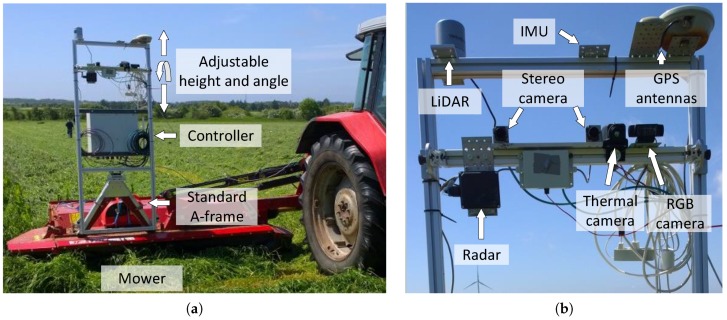
Sensor frame including the controller (**a**). Sensors on the sensor platform (**b**). Figure taken from [34].

Images are recorded using a stereo camera composed of two Flea 3 GigE color cameras (Model: FL3-GE-28S4C-C, Point Grey Research Inc, Richmond, Canada) with a global shutter, a resolution of 1920 × 1080, a baseline of 24 cm and a frame rate of 15 Hz. The stereo camera is mounted on a sensor platform [33,34] roughly 2 m above the ground; see Figure 2. The algorithm uses images taken from the left camera, and the data from the right camera are only used for estimating the distance to obstacles when evaluating the proposed algorithm.

**Figure 3:**
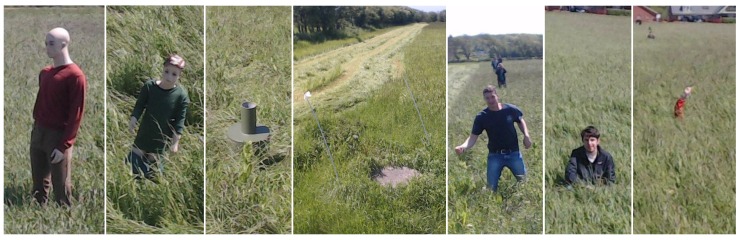
Two mannequins, a barrel, a well and three people, one only showing his arm, in the field.

**Figure 4:**
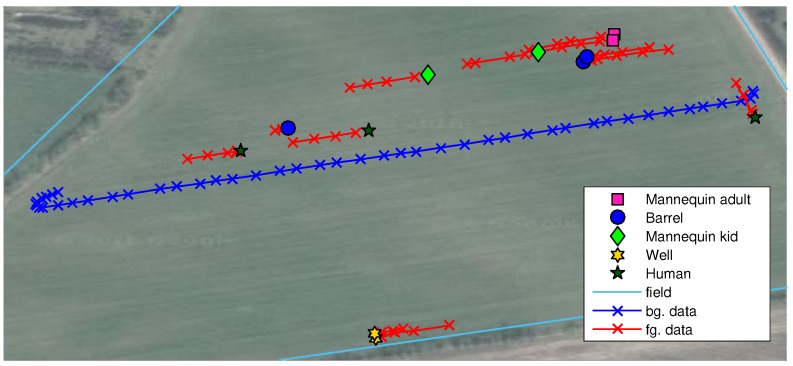
Obstacles and tractor image positions for background data (blue) and test data (red). Orthophoto from Google Maps.

Images were recorded during a grass mowing operation in a 7.5-ha grass field near Lem, Denmark, in June 2015 on a sunny and partly cloudy day. To simulate potential obstacles humans, green barrels [35], kid and adult mannequins were placed in the trajectory of the tractor. Image examples are presented in Figure 3. Two datasets are used in this work; background data for generating the background model and test data for evaluating anomaly detection configurations. The background data consist of 56 images recorded in a single crossing of the field from one end to the other. The test data are a selection of 48 images from 12 scenarios with per-pixel annotations of humans, houses, mannequins, barrels and wells. Each scenario contains 3–5 image samples taken in a range of 2–20 m to the obstacle as the tractor approaches. The obstacles and the tractor positions are estimated using GPS measurements and depicted in Figure 4. Other obstacles visible by the camera, such as humans watching the experiment, shelter belt and a house, are not depicted in the figure.

## 3. Methods

The anomaly detection framework combines BS methods with high-level features, extracted from convolutional layers in a CNN. The high-level features make the BS robust to changes caused by camera motion and sensitive to new content or elements that are unnatural in an agricultural field.

### 3.1. CNN Features for Anomaly Detection

**Figure 5:**
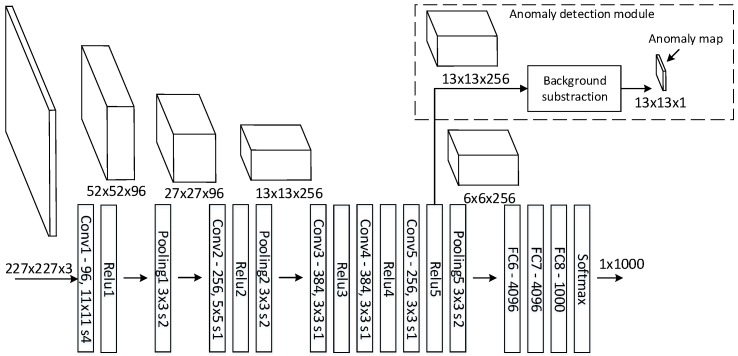
Caffe reference model [36] and the anomaly detection module. The notations of, e.g., “Conv1—96, 11 × 11 s4” mean that convolutional layer Conv1 has 96 kernels with a receptive field of 11 × 11 and a stride of four.

Figure 5 depicts how the anomaly detection framework uses features from a CNN. The Caffe reference [36] model, a variation of AlexNet [9], forward passes a fixed sized image through the network, generating intermediate features maps, depicted by cubes. The final feature map (6 × 6 × 256) is forwarded through multiple fully-connected neural networks (FC) and a softmax layer generating a prediction vector (1 × 1000). For a CNN trained on ImageNet, the prediction vector contains a value for each of the 1000 object types, which is standard in the ImageNet classification task [25]. A feature map describes the characteristics of the input image, where each channel corresponds to a specific feature. In the first convolutional layer, channels will activate on low level features, such as edges, blobs and colors. In deeper layers, channels will activate on high level features with more abstract characteristics, such as faces, text or vehicles [37].

The figure illustrates how feature map dimensions decrease through the network going from an input image of 227 × 227 to a 6 × 6 feature map. Each entry in the 6 × 6 features map contains 256 high level features describing an area (receptive field) of 195 × 195 in the original image for every 32 pixels.

The anomaly detection module uses feature maps from a sequence of images to model the background. With a background model, it is possible to describe the distance from a feature map entry to the background model. As depicted in Figure 5, a feature map of 13 × 13 entries will generate an anomaly map of 13 × 13.

Many state-of-the-art deep learning-based detectors generate feature maps that can be used by an anomaly detection module. Thus, existing CNN based detector are able, to add the anomaly detection module for a small computational cost, to detect anomalies.

**Figure 6:**
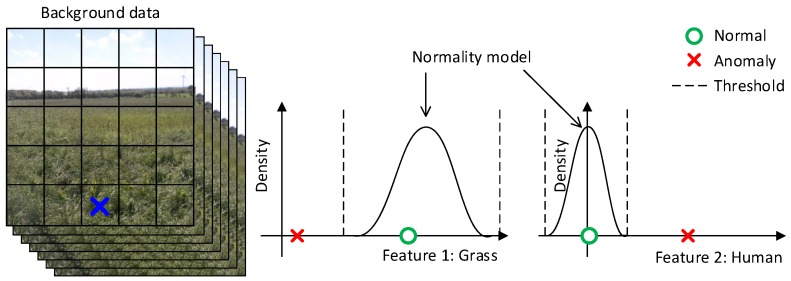
Representation of the background subtraction module for only a single feature map entry and two features.

Figure 6 illustrates the intuition behind the background subtraction module. At the left, feature maps are calculated for a sequence of images. The feature map for an image is represented with a grid, where each feature map entry describes an area in the original image with, e.g., 256 features for the Caffe reference model. For simplicity, only a single feature map entry (marked with a blue cross) and two features are used in this illustration. At the middle and right, the feature map entry is modeled with a Gaussian distribution for respectively a grass-like and a human-like feature and shows that the normality model generally expects high values for grass-like features and low values for human-like features. The normality model is then based on some threshold able to detect an outlier or anomaly.

#### 3.1.1. Mapping of Feature Maps back to the Input Image

**Figure 7:**
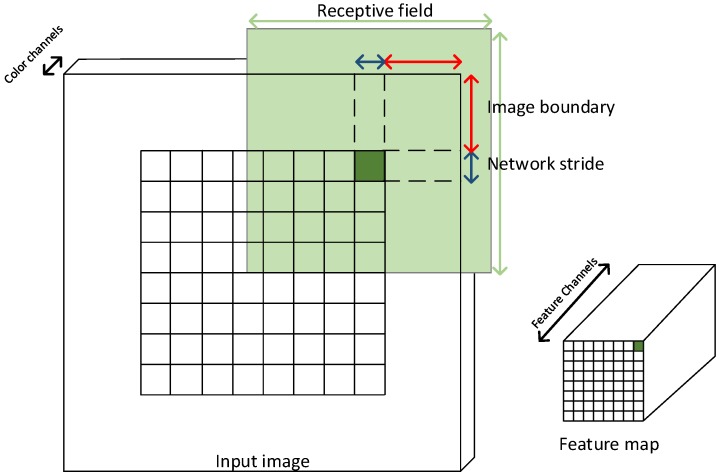
Mapping of a feature map entry (marked with dark green) back to the input image. A feature map entry describes an area of similar size as the network stride in the input image. A feature is determined by the information captured in the receptive field. Image boundary areas are not explicitly described by a feature map entry; only implicitly by the receptive field of nearby feature map entries.

To determine what a feature map or an anomaly map entry corresponds to in the input image, the network stride, network receptive field and image boundary must be determined as illustrated in Figure 7. The network stride is the pixel spacing between network predictions. The network receptive field is the area a feature map entry describes in the original image. Valid convolutions will create an undefined area along the image border. In Figure 7, this is defined as the image boundary. The receptive field of a prediction may use the image boundary to provide some implicit description of the undefined area as illustrated in Figure 7. To compare anomaly detection with the ground truth, ground truth images are cropped by the image boundary, and the anomaly detection map is resized by the network stride using nearest neighbor interpolation.

#### 3.1.2. Network Modifications

To target the network for anomaly detection, a few modifications of the Caffe reference CNN architecture is performed. The low 6 × 6 resolution of the final feature map provides poor spatial resolution in the resulting anomaly map. The nature of convolutional and subsampling layers allows larger (in height and width) images to be forwarded through the network and generates higher resolution feature maps. However, unlike convolutional and max-pooling layers, the FC and softmax layers require a fixed sized image. By removing the softmax, the three FC and the final max-pooling layer, the network is able to double the feature map resolution and process larger images. Additional advantages of removing the final layers are a faster forward time and a much lower memory footprint, that is critical for an embedded GPU with limited memory and computation power. For VGG16 on a Titan X GPU, the forward pass drops 39.5% from 20.5 ms to 12.4 ms, and the memory footprint drops 74.6% from 1485 MB to 376 MB. For the Caffe reference model, the forward pass drops 36.8% from 3.75 ms to 2.37 ms, and the memory footprint drops 78.9% from 303 MB to 64 MB.

An unwanted effect of zero-padded convolutions is that feature maps get corrupted or become invalid along the image border. In image classification, this is not critical, as the object of interest is placed in the image center. In anomaly detection, features are required to be valid in all image positions. This is handled by only using valid convolutions or no zero-padding for all layers in a network. Changing between valid and invalid convolutions will, for a network with no FC-layers, not require a network to be retrained.

#### 3.1.3. Network Feature Map Investigation

A range of feature configurations are tested.

**Figure 8:**
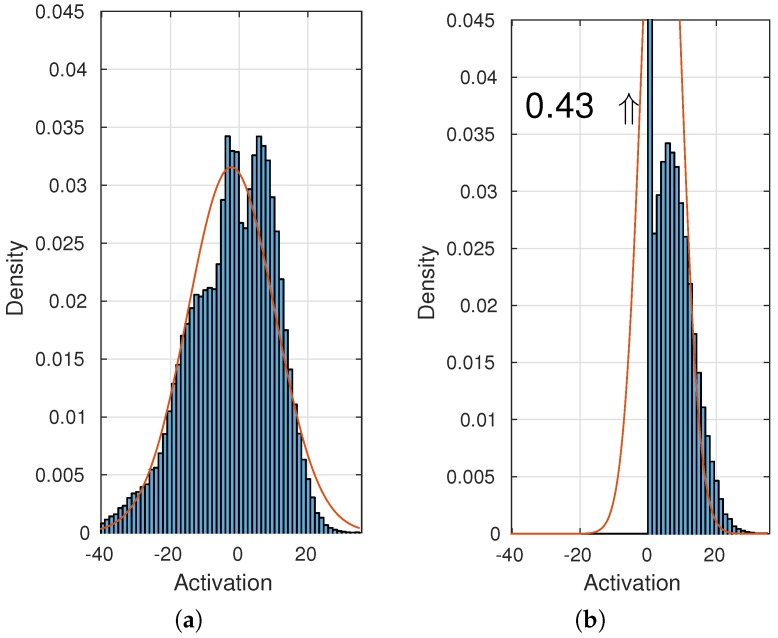
Histogram of a neural network unit before (**a**) and after (**b**) a ReLU (Rectified Linear Unit).

1. Use features from both the Caffe reference model (AlexNet) and the VGG architecture.
2. Use features from different layers in a network. Earlier layers are more general [38,39], require less computation and provide higher feature map resolution.
3. Use features before the activation function ReLU (Rectified Linear Unit). A Gaussian distribution will more accurately resemble the output of a convolutional layer before the ReLU, as depicted in Figure 8.
4. Use dilated convolutions as described in [40] to double the feature map resolution for a given input image without doubling the input image size. The feature map is increased by removing max-pooling layers and doubling the dilation factor in subsequent layers.
5. Append an 1 × 1 convolutional layer before the final max-pooling layer to perform feature compression or dimension reduction as in GoogLeNet [11]. The high number of features provided by a CNN, e.g., 256 by AlexNet in the final convolutional layer, makes the computational complexity of the background model high. To avoid retraining a network from scratch with fewer features, a 1 × 1 convolutional layer is appended to an ImageNet pre-trained network. Three network architectures are created with respectively 128, 64 and 32 kernels for the appended 1 × 1 layer and fine-tuned on ImageNet.

### 3.2. Image Model Geometry

**Figure 9:**
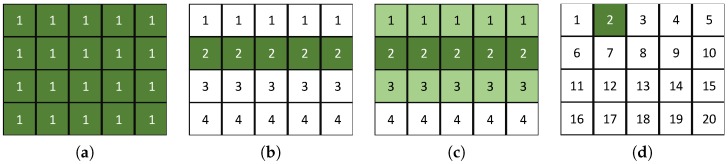
Image model geometries. (**a**) Geometry 1; (**b**) Geometry 2; (**c**) Geometry 3; (**d**) Geometry 4.

In conventional background subtraction algorithms [41], each pixel is classified as either foreground or background using a model of the background. As illustrated in Figure 6, a normality model is typically generated for each feature map entry over a sequence of images using only features from the same image position. For a front-facing camera in an agricultural field, the image is expected to have a specific geometry as depicted in Figure 1. We investigate various image modeling geometries:

1. Single model: Models the whole image using a single model. The model uses all feature map entries in the image over a sequence of images. As illustrated in Figure 9a, a single model is generated for a 4 × 5 feature map.
2. Row model: Models each row in the image. Each model uses feature map entries from the current row over a sequence of images. As illustrated in Figure 9b, four models are generated for a 4 × 5 feature map.
3. Extended row model: Models each row in the image. Each model uses feature map entries from the current and neighboring rows over a sequence of images. As illustrated in Figure 9c, four models are generated for a 4 × 5 feature map.
4. Traditional BS model: Models each entry in a feature map. Each model uses only the current feature map entry over a sequence of images as the traditional background subtraction algorithm [41]. As illustrated in Figure 9d, 20 models are generated for a 4 × 5 feature map.

### 3.3. Normality Model Types

**Figure 10:**
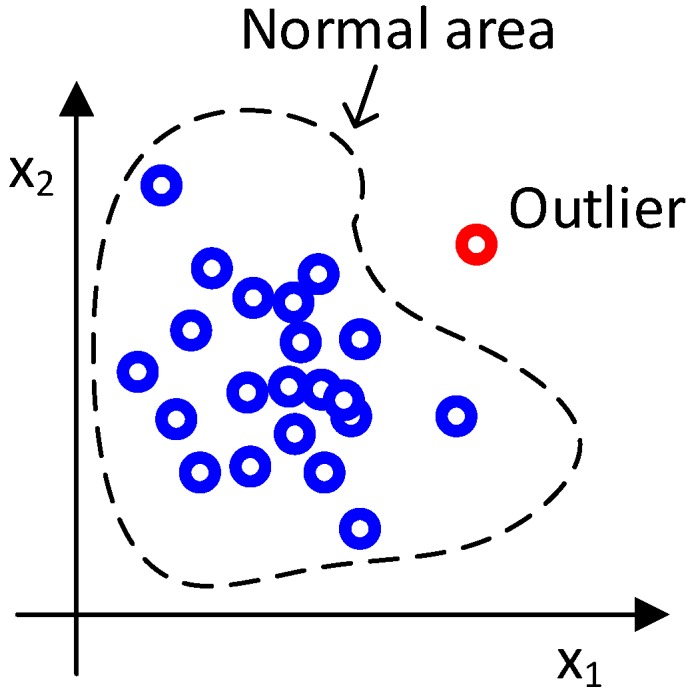
Outlier detection.

An outlier detector uses a background model or a normality model to model the background data. Outliers are defined as a sample outside the normal area. The normal area is defined by the normality model, background data and a threshold; see Figure 10 for two-dimensional examples.

A feature sample is defined as an entry from a feature map with *D* features. A normality model is generated from *N* feature samples. We denote background feature samples for generating a normality model by X; hence, X is a D×N matrix with *N* samples with *D* features. The column *j* of X, defined as $x_{j}$, is all of the features for a sample *j*.

Feature samples are gathered over a sequence of images. However, depending on the model geometry, background samples are gathered from either the whole image, feature map rows or only specific feature map entries. In a conventional background subtraction, a “traditional BS model”, a model uses only samples from a specific image position over a sequence of images. In image model Geometry 1, a model uses all samples in all positions in a sequence of images. The origin of feature samples, in terms of the feature map entry and image, are ignored in the following section and simply denoted by X.

The aim of an outlier detector is to determine an anomaly measure, denoted by $M\left(\tilde{x}|\theta \right)$. $M\left(\tilde{x}|\theta \right)$ measures the distance between a sample, $\tilde{x}$, with an unknown class and the normality model with parameters ***θ***. The model parameters are dependent on the normality model type and background samples. When $M\left(\tilde{x}|\theta \right)$ is larger than a specified threshold, $\tilde{x}$ is classified as an outlier/abnormality. The threshold value is selected based on the annotated test data. We address this issue in the result sections.

#### 3.3.1. Mean and Median

The mean parameters $\theta_{mean}=\mu$ are the mean value of each feature *i* over all background samples $\mu ={\left[\mu_{1}\cdots \mu_{i}\cdots \mu_{D}\right]}^{T}$. For a sample, $\tilde{x}$, the anomaly measure is defined as the Euclidean distance between the mean value of a feature to a sample feature.

(1) $$M_{\mathrm{mean}}\left(\tilde{x}|\mu \right)=∥\mu -\tilde{x}∥=\sqrt{\sum_{i=1}^{D}{\left(\mu_{i}-{\tilde{x}}_{i}\right)}^{2}}$$

For a median model, the median value m is used instead of the mean value.

(2) $$M_{\mathrm{median}}\left(\tilde{x}|m\right)=∥m-\tilde{x}∥$$

#### 3.3.2. k-NN

The kNN model [4] parameters $\theta_{\mathrm{kNN}}=X$ consist of all background samples, X. The anomaly measure is the Euclidean distance from the k-nearest neighbor sample $x_{\mathrm{kNN}}$ to a sample $\tilde{x}$.

(3) $$M_{\mathrm{kNN}}\left(\tilde{x}|X\right)=∥x_{\mathrm{kNN}}-\tilde{x}∥$$

#### 3.3.3. Single Variate Gaussian

The single variate Gaussian (SVG) [41] model parameters $\theta_{\mathrm{SVG}}=\left(\mu ,\sigma^{2}\right)$ comprise the mean value, ***μ***, and the variation for each feature *i* taken over all samples in the training data $\sigma^{2}={\left[\sigma_{1}^{2}\cdots \sigma_{i}^{2}\cdots \sigma_{D}^{2}\right]}^{T}$. The anomaly measure is defined as the Mahalanobis distance between a feature sample and the single variate Gaussian distribution along each dimension.

(4) $$M_{\mathrm{SVG}}\left(\tilde{x}|\mu ,\sigma^{2}\right)=∥\left(\tilde{x}-\mu \right)⊙\frac{1}{\sigma }∥=\sqrt{\sum_{i=1}^{D}\frac{{\left(\tilde{x_{i}}-\mu_{i}\right)}^{2}}{\sigma_{i}^{2}}}$$

Unlike the multivariate Gaussian model described in the next sections, feature dimensions are treated independently (Σ is a diagonal matrix).

#### 3.3.4. Multivariate Gaussian

The multivariate Gaussian (MVG) [41] parameters $\theta_{\mathrm{MVG}}=\left(\mu ,\Sigma \right)$ comprise the mean value, ***μ***, and the covariance matrix, **Σ**.

(5) $$\Sigma =\frac{1}{N-1}\sum_{j=1}^{N}\left(x_{j}-\mu \right){\left(x_{j}-\mu \right)}^{T}$$

The anomaly measure is defined as the Mahalanobis distance between a sample $\tilde{x}$ and the Gaussian distribution.

(6) $$M_{\mathrm{MVG}}\left(\tilde{x}|\mu ,\sigma^{2}\right)=\sqrt{{\left(\tilde{x}-\mu \right)}^{T}\Sigma^{-1}\left(\tilde{x}-\mu \right)}$$

#### 3.3.5. Gaussian Mixture Model

The Gaussian mixture model (GMM) [3] parameters $\theta_{\mathrm{GMM}}=\left(\mu_{1}\cdots \mu_{M},\Sigma_{1}\cdots \Sigma_{M}\right)$ comprise M Gaussian models with a mean value, $\mu_{i}$, and the covariance matrix, $\Sigma_{i}$, for each model *i*. Models are determined using expectation-maximization [42]. The anomaly measure is defined as the Mahalanobis distance between a sample and the nearest neighbor Gaussian model, $\theta_{\mathrm{NN}}=\left(\mu_{\mathrm{NN}},\Sigma_{\mathrm{NN}}\right)$.

(7) $$M_{\mathrm{MVG}}\left(\tilde{x}|\mu_{\mathrm{NN}},\Sigma_{\mathrm{NN}}\right)=\sqrt{{\left(\tilde{x}-\mu_{\mathrm{NN}}\right)}^{T}\Sigma_{NN}^{-1}\left(\tilde{x}-\mu_{\mathrm{NN}}\right)}$$

### 3.4. Implementation Details

CNN models are executed using Caffe, a framework for deep learning [36] and the Caffe-MATLAB interface allowing MATLAB to use CNN features from network layers. The background models and the evaluation of various configurations are implemented in MATLAB. The original images with a resolution of 1080 × 1920 are cropped by 700 pixels to remove the tractor from the left side of the image. Images are resized by a factor of 0.75 and cropped slightly again to form valid dimensions for a CNN network.

## 4. Results

Results are divided into three subsections. The first subsection shows trends across many network configurations. A specific configuration is not optimal across any image geometric modeling, normality model or output layer, e.g., the optimal normality model type depends on the network layer and the geometric modeling. The first sections are intended to show configuration trends across the vast number of configurations. The second subsection is targeted directly at reaching the most optimal configuration in terms of accuracy and computation time. The third subsection compares a top performing configuration with state-of-the-art object detection algorithms.

**Figure 11:**
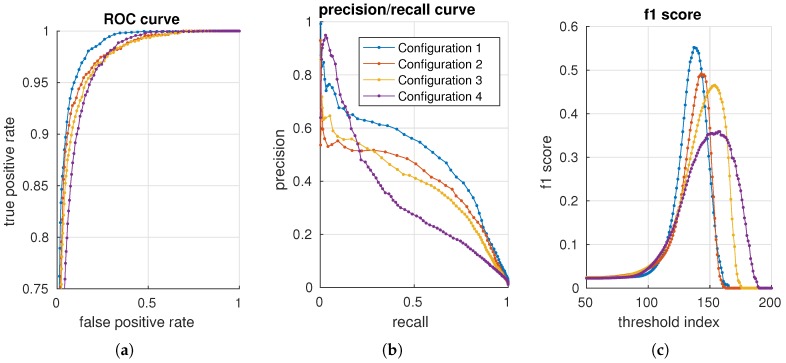
Illustration of an ROC curve (**a**), a precision/recall curve (**b**) and f1 scores (**c**) for four configurations.

We use the background data to create a normality model for all configurations. Each configuration is evaluated against the per-pixel annotated test data. In total, 460 configurations are evaluated. For each configuration, a receiver operating characteristic (ROC) [43] curve, precision/recall (PR) [44] curve and the f1 score [45] are generated with 200 distributed thresholds. Four configurations are presented in Figure 11 using, respectively, an ROC, precision/recall and an f1 score curve. The advantage of precision/recall and the f1 score is that true negatives are not included in the metric. As the data mostly contain negative samples (field samples), the (zoomed) ROC plot shows curves that are squeezed together.

To get a single valued accuracy measure, for each configuration and metric, the maximum f1 score, area under the curve for an ROC (AUC_ROC) curve and the area under the curve for a precision/recall (AUC_PR) curve are used in the following sections.

### 4.1. Trends Across Configurations

**Figure 12:**
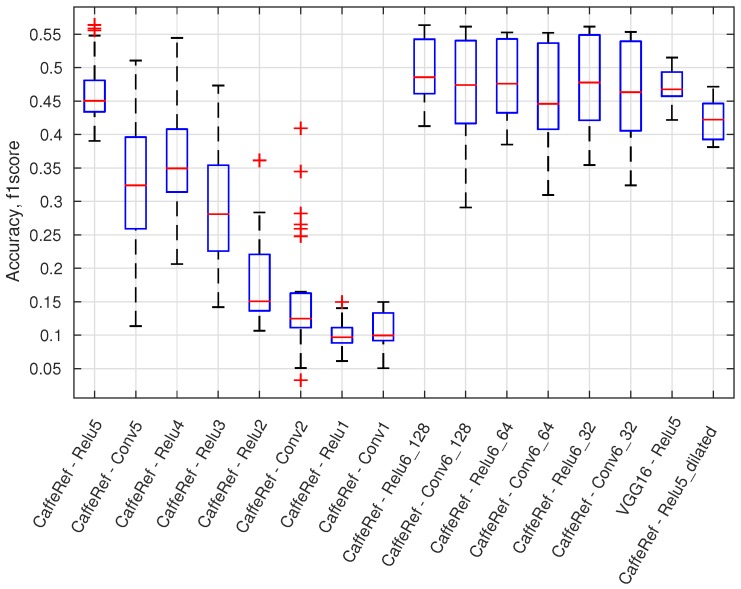
Accuracy (f1 score) for a given output layer across all model normality types and image model geometries.

This sections provides an overview of the 460 configurations by using the maximum f1 score and boxplot presentations. In Figure 12, a boxplot presents the accuracy variation (maximum f1 score) for all configurations using a specific output layer. There are, e.g., 56 different configurations for the CaffeRef—Relu5 output layer. Generally, the accuracy degrades for lower feature layers; dilated layers are not preferable, and ReLU layers are mostly preferred over convolutional (Conv) layers. Generally, feature compression using 1 × 1 convolutional layers (Relu6_X and Conv6_X) does not significantly reduce performance. Compression of features will reduce the processing time for an anomaly module and will for some classifiers also improve accuracy.

**Figure 13:**
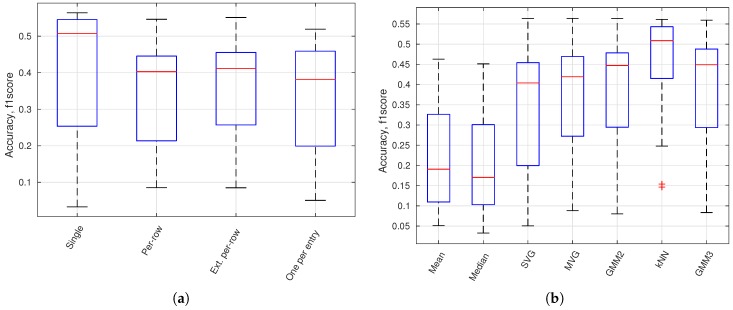
Accuracy for model geometries (**a**) and normality models (**b**). (**a**) Accuracy (f1 score) for all image model geometries; (**b**) accuracy (f1 score) for all normality model types.

In Figure 13a, a boxplot presents the accuracy variation for all configurations using a specific image model geometry. It shows that a single model or the model per-row performance is better than the traditional background subtraction geometry with one model per entry. In Figure 13b, a boxplot presents the accuracy variation for all configurations using a specific normality model type. GMM 2 and GMM 3 is a GMM model with respectively 2 (M=2) and 3 (M=3) Gaussian models. The kNN-based model is of highest performance followed by the Gaussian-based models. Mean and median models are inferior to other model types.

### 4.2. Determining the Best Set of Configuration

**Figure 14:**
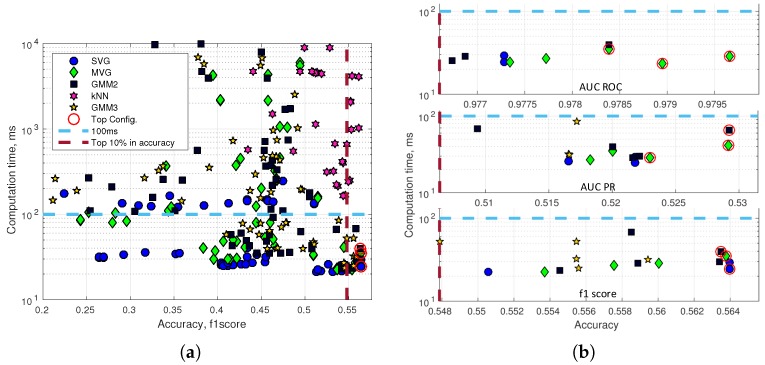
Accuracy and computation time. (**a**) Accuracy is measured using the f1 score; (**b**) top configurations rectangle of AUC_ROC, AUC_PR and the max f1 score.

The optimal configuration is a combination of high accuracy and speed performance, e.g., the kNN classifier generally has a high accuracy performance. However, even the fastest kNN configuration has a computation time of more than 200 ms for a single image. Figure 14a shows the computation time for a single image versus the f1 score accuracy performance. Two lines draw a top configuration rectangle with the fastest (<100 ms) and the highest accuracy (top 10%) anomaly detectors. Figure 14b (a top performing SVG in the bottom plot of Figure 14b is ignored as the SVG just below has identical accuracy) presents the top configuration rectangle for respectively AUC_ROC, AUC_PR and the maximum f1 score. Feature calculations are performed on a GTX Titan X 12GB Maxwell architecture, and the anomaly detection module is executed on a Intel Xeon 2.1 GHz six-core CPU (E5-2620V2). The three configurations with the highest accuracy of each plot are marked with a red circle.

**Figure 15:**
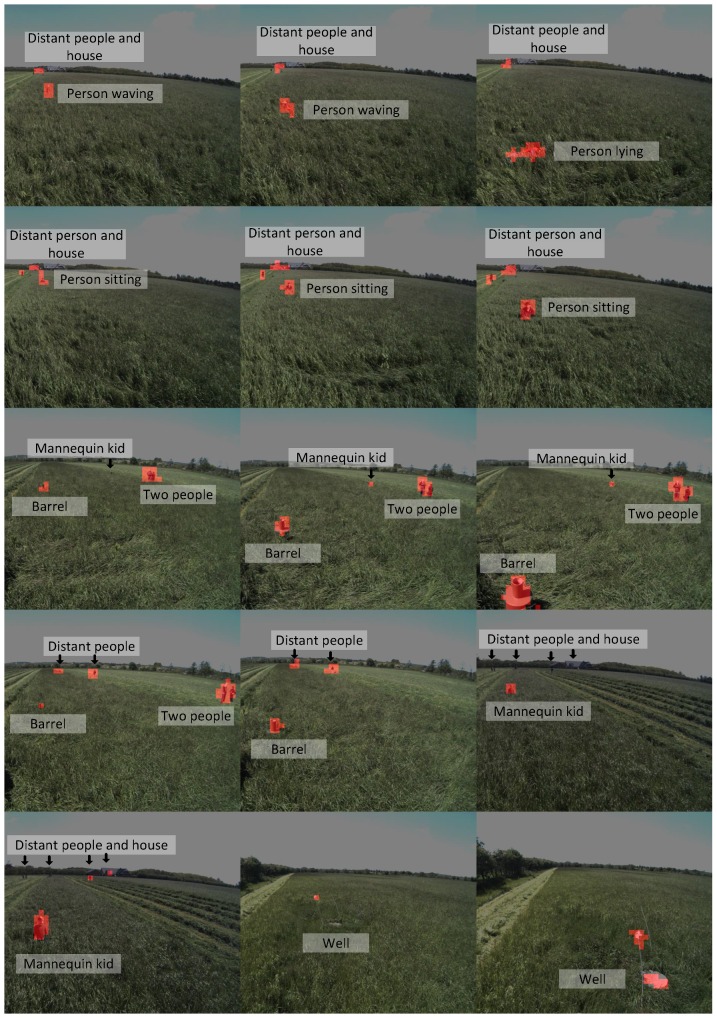
DeepAnomaly detections. No false positives are present in the images.

The seven unique top performing configurations have been listed in Table 1; two of the nine configurations have a duplicate. The computation time is listed for a prediction of a single image including feature calculations (Total Pred.), a prediction of a single image without feature calculations (Model Pred.) and for updating the background model (Model Update). The model update is not expected to be performed for every image and is therefore not considered as time critical as the total prediction time for an image. The anomaly detection module can use features from another CNN-based detector and avoid the computational cost of computing its own features. The model prediction time is listed to show the computation cost of adding anomaly detection to an existing CNN-based detector. Apart from Number 2, the top configurations are very similar in accuracy. Generally, the table includes only Gaussian-based normality models and single model-based image model geometry. The slightly faster and simpler Configuration 6 is in this paper defined as DeepAnomaly and used in future experiments. DeepAnomaly has a total prediction time of 25 ms (40 FPS), a model prediction time of only 4 ms and a model update time of 834 ms. The performance of DeepAnomaly is presented in a set of image examples in Figure 15. The pixel accuracy on the annotated test data is used for selecting a threshold.

### 4.3. Object Detection vs. Anomaly Detection

The accuracy of anomaly configurations has been reported using the f1 score and AUC measures, allowing anomaly configurations to be compared mutually. However, such accuracy measures provide only an indication of the performance of DeepAnomaly compared to other state-of-the-art detectors. In this section, a quantitative evaluation metric is defined to evaluate DeepAnomaly with state-of-the-art detection algorithms.

The comparison is challenged by the inconsistent outputs of the selected algorithms. Algorithms may either detect one or multiple object types, and the location of objects are marked with either a bounding box or per-pixel predictions. To solve this inconsistency problem, a detector is only evaluated for its ability to detect them (other annotated obstacles are ignored). Humans are used as test objects, as all algorithms are able to detect humans. Ground truth annotations, which are per-pixel annotations of obstacles, are converted to bounding boxes and extended by 12 pixels on all sides. A detection is true (true positive) when the detection area overlaps an annotated human by more than 50%. An overlap of less than 50% is a false detection (false positive), unless the detection overlaps an annotated non-human obstacle by more than 50%. A false negative is defined as a human annotation that has not been detected.

**Figure 16:**
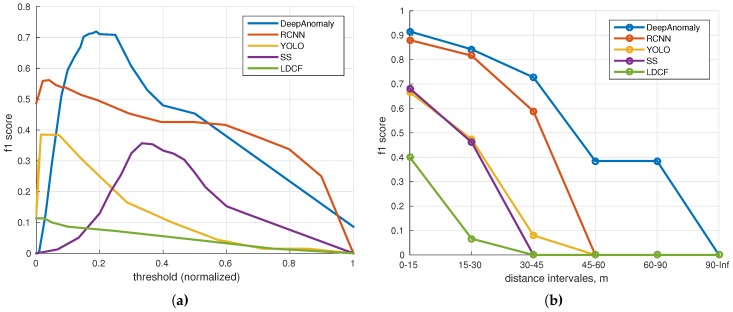
Accuracy performance (f1 score) of DeepAnomaly and four state-of-the-art object detection algorithms. (**a**) Accuracy relative to thresholds; (**b**) accuracy relative to distance intervals.

Four object detection algorithms have been selected: a pedestrian detector “local decorrelated channel features” [46,47,48] (LDCF) trained on INRIA (Institut national de recherche en informatique et en automatique) Person Dataset ; two deep learning multi object detection algorithms “you only look once” [18] (YOLO) and “faster R-CNN” [16] (RCNN), trained on ImageNet and Pascal VOC (Visual Object Classes)[24]; one semantic segmentation algorithm “fully convolutional neural networks for semantic segmentation” [19,49] (SS) trained on ImageNet and Pascal Context [50]. Figure 16a shows the f1 score for each algorithm sweeping over a set of thresholds. The highest f1 score is achieved by DeepAnomaly (0.720) followed by RCNN (0.562), YOLO (0.385), SS (0.357) and LDCF (0.113).

The normality model of DeepAnomaly is sensitive to the used background samples; meaning that the optimal threshold may change for each model update. However, DeepAnomaly forms a little plateau of top accuracies in the range of 150–250 (0.15–0.25 normalized), showing that the threshold is partly robust to model updates.

Figure 16b shows the f1-score at different distances using the optimal threshold for each algorithm. DeepAnomaly is able to detect humans at longer distances and is either of similar or better performance on short ranges using a smaller CNN model than RCNN, YOLO and SS. Table 2 compares the algorithms’ accuracy, computation time, the number of model parameters (# Model Params) and its ability to classify obstacles (Class.) and detect unknown obstacles (Unk. Types). (The number of model parameters of RCNN, YOLO and LDCF is not directly specified in the respective publications. The number for RCNN only includes parameters of convolutional layers (regression and classification modules parameters are ignored). The number for YOLO is based on the described model. The number for LDCF is a rough estimate.). DeepAnomaly detects humans with better accuracy at longer distances in real time. RCNN shows similar performance on shorter distances. However, the computation time of RCNN is unsuited for real-time applications. A qualitative test is presented in Appendix A, showing detections from all algorithms in 30 images containing humans. A key feature of DeepAnomaly is the ability to detect unknown objects/anomalies and not just a set of predefined objects. Secondly, DeepAnomaly uses only a pre-trained network and does not require the time-consuming task of making algorithm/object-specific training data. The high accuracy, the low number of model parameters and the low compute time make DeepAnomaly suited for a real-time detection system on an embedded GPU. The drawback of DeepAnomaly is that no label or classification is provided for each detection.

## 5. Discussion

Deep learning-based object detection and semantic segmentation have recently showed state-of-the-art results in detecting specific objects. However, in an agricultural context, they have difficulty in detecting heavily occluded and distant objects, and methods are, by definition, trained to recognize a predefined set of object types. DeepAnomaly can exploit the very homogeneous characteristics of an agricultural field to detect distant, heavy occluded and unknown objects. Qualitatively, this is illustrated in Figure 15, where DeepAnomaly detects a distant and occluded mannequin kid, a human showing only his arm, a heavy occluded olive-green barrel (with similar color as the field), a well cover and detections of obstacles with a size of less than 16 × 16 pixels. By using DeepAnomaly in junction with other deep learning algorithms, it can save computations by using convolutional features from other networks. DeepAnomaly also spares the time-consuming task of providing domain- or algorithm-specific annotated data.

A detection metric for detecting humans is defined to compare DeepAnomaly with four state-of-the-art algorithms. The comparison shows that DeepAnomaly is better at detecting humans at longer ranges (45–90 m). RCNN has similar performance at short range (0–30 m). However, with much fewer model parameters and a (182 ms/25 ms=) 7.28-times faster processing time per image, DeepAnomaly is more suitable for real-time applications running on an embedded GPU. The used detection metric copes with dissimilar outputs of the evaluated algorithm and will not favor a precise localization/position of a detection. However, in the context of autonomous vehicles in agriculture, the exact bounding box position or semantic segmentation at pixel-level precision is not of critical importance. Rough localization markings (±12 pixel) are sufficient, and more important is the detector’s ability to, in real time, detect obstacles even when they are heavily occluded, distant and potentially unknown. However, it is important to state that DeepAnomaly requires specific conditions in terms of visually-homogenous surrounding and a low incidence of anomalies. This is not a limitation for YOLO, RCNN, SS and LDCF.

Evaluating algorithms that are trained on different data is basically unfair; especially for LDCF, which uses a much smaller dataset. However, YOLO, RCNN, SS and DeepAnomaly use parameters from a network trained on ImageNet, including one additional dataset with algorithm-specific annotations (bounding boxes, per-pixel annotations, background data). One may argue that DeepAnomaly has an (unfair) advantage, as it learns a background model from data recorded in the same field as the test. This is true for a classification problem where test and training must be uncorrelated. However, for background subtraction algorithms, it is a basic concept that an algorithm learns the characteristics of a specific setting to better detect foreground elements. Similar for DeepAnomaly, the algorithm is intended to exploit and learn the characteristics of a particular field to better detect anomalies.

DeepAnomaly does not provide labels as an obstacle detection algorithm. However, by using DeepAnomaly as a region proposal algorithm, labels can be given by forwarding anomalies through a classification network. This is related to RCNN and other deep learning object detection algorithms [14,15,16,17] that initially use a region proposal algorithm providing between 300 and 2000 regions per images. Each region is then forwarded through a classification network providing a label for each region. DeepAnomaly can be used as an effective region proposal algorithm providing only a few or no regions per image.

The normality model must be updated regularly without including foreground elements. This difficulty is partly solved in an agricultural context where the incidence of anomalies is very low. Secondly, the experiment shows that the initial model generalizes to many positions in the field, meaning that the model does not require very frequent updates. Furthermore, foreground elements can be filtered out by other obstacle detections algorithms. For, e.g., RCNN, the anomaly algorithm should not include feature map entries in the background model that is inside an RCNN bounding box detection. In future work, we are interested in extending the anomaly framework to other sensor modalities. Depth sensors are able to detect obstacles that protrude from the crop surface, and a thermal sensor can detect outlier heat radiations in the field. The advantage of combining visual, depth and thermal modalities is that anomalies are more independent and described by physically different characteristics, making it unlikely for foreground/non-field obstacles to be included in the background model (unless the foreground element has similar visual appearance, height and temperature as the crop).

The robustness of threshold values and procedures for doing model updates are addressed, but not implemented in actual experiments. This paper is focused on practical considerations for using deep learning features and elaborated investigations. The investigation comprises a total of 460 settings that are evaluated in terms of processing time and accuracy, using three different accuracy metrics. A top performing configuration is then compared to state-of-the-art detection algorithms for their ability to detect humans in general and at different range intervals.

## 6. Conclusions

This work illustrates that a background subtraction algorithm can be used successfully for a non-static camera in agriculture by using high level features from a deep convolutional neural network. An elaborated investigation has been conducted on a broad set of configuration to determine a high performing setting for an anomaly detection system. This configuration is named DeepAnomaly. It is a simple algorithm that exploits the homogenous characteristics of an agricultural field, i.e., detect heavily occluded, distant and unknown objects without the time-consuming task of providing algorithm- and object-specific training data. DeepAnomaly is foremost an anomaly detector. However, it has shown comparable or better results for obstacle detection in an agricultural context. It is able to detect humans better and at longer distances than state-of-art networks with 40 FPS using less training data and a smaller network. The low computation time and low memory footprint make it suited as a real-time system and for embedded GPUs. DeepAnomaly is also able to assist an existing deep learning detection system by using the existing feature maps. Thus, for only a small computational cost, 4 ms on a CPU, a CNN-based detector can be extended to also detect distant, heavily occluded and unknown obstacles.

multiple

## Figures and Tables

**Table 1:** Shows seven top performing configurations in terms of computation time and three accuracy measures.

Nr	Classifier	Metric	Model Area	Layer Output	ROC AUC	PR AUC	F1 Score	Computation Time (ms)		
								Total Pred.	Model Pred.	Model Update
1	MVG	ROC/PR	Single	Conv6_128	0.980	0.523	0.560	29	8	1,219
2	MVG	ROC	Single	Conv6_64	0.979	0.476	0.537	23	4	438
3	MVG	ROC/f1	Single	Relu5	0.978	0.520	0.564	35	14	6,360
4	MVG	PR	Single	Relu4	0.970	0.529	0.544	42	21	12,169
5	GMM2	PR	Single	Relu4	0.969	0.529	0.536	66	42	142,727
6	SVG	f1	Single	Relu5	0.977	0.522	0.564	25	4	834
7	GMM2	f1	Single	Relu5	0.978	0.520	0.564	40	19	5,325

**Table 2:** Comparison of of DeepAnomaly with four state-of-the-art algorithms for obstacle detection. YOLO, you only look once; SS, semantic segmentation; LDCF, local decorrelated channel features.

Name	F1 Score	Compute Time (ms)	Compute Unit	Class.	Range	Unk. Types	Object Specific Training Data	# Model Params.
DeepAnomaly	0.720	25	GPU + CPU	No	Far	Yes	No	3.7 M
RCNN	0.562	182	GPU	Yes	Mid	No	Yes	14.7 M
YOLO	0.385	23	GPU	Yes	Low	No	Yes	262 M
SS	0.357	237	GPU	Yes	Low	Yes	Yes	134 M
LDCF	0.113	348	CPU	Yes	Low	No	Yes	<0.01 M

**Figure A1:**
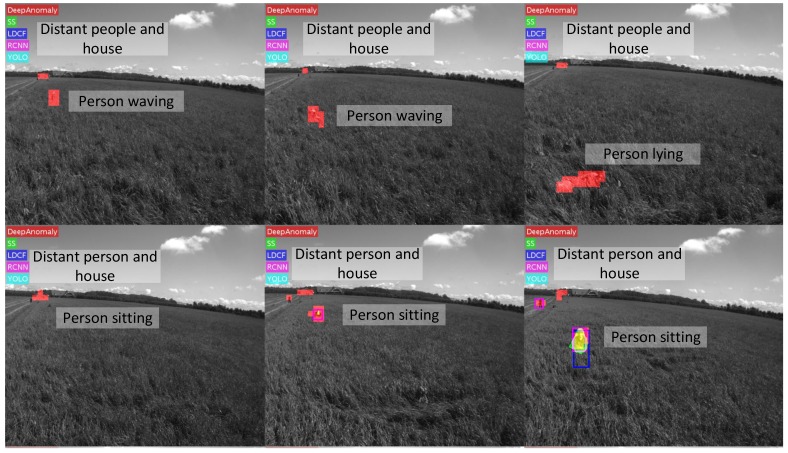

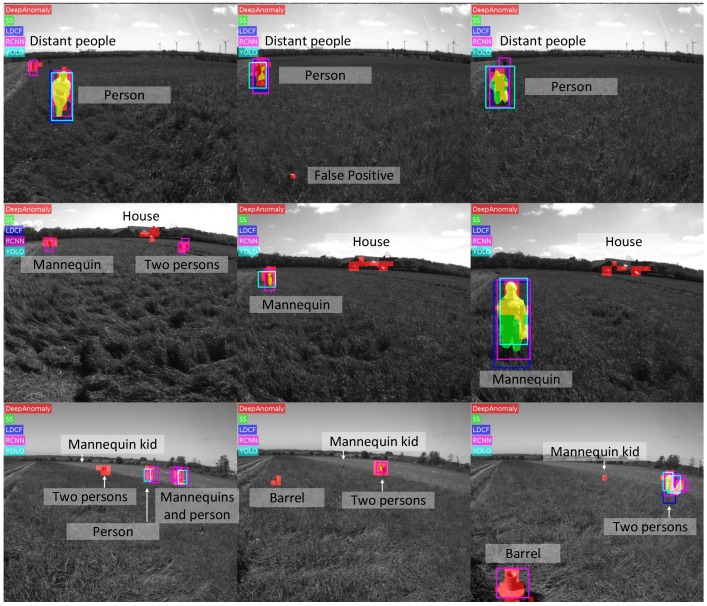
Detection examples of 15 image with humans.

**Figure A2:**
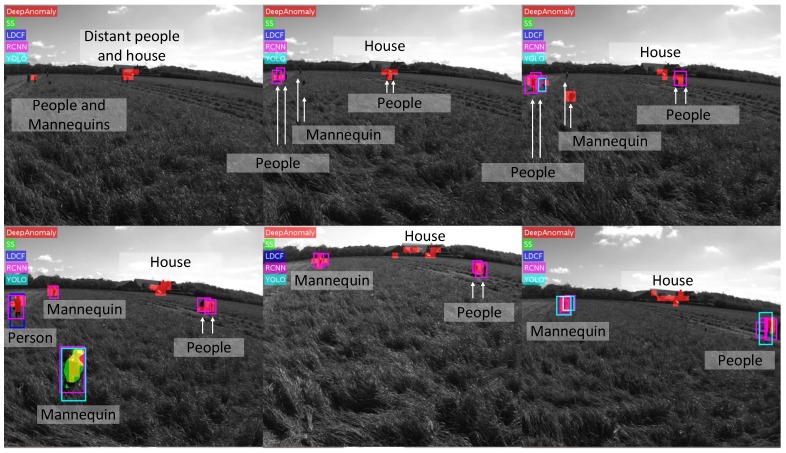

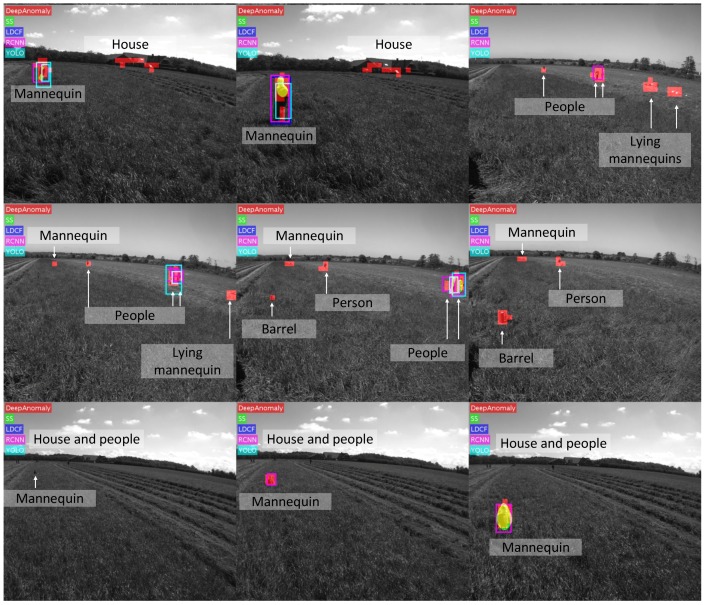
Detection examples of 15 image with humans.

## Appendix A

Figure A1 and Figure A2 show detections by DeepAnomaly, RCNN, SS, YOLO and LDCF for 30 image samples containing humans. Each detector is presented with a specific color. Detections by RCNN, YOLO and LDCF are presented with a bounding box. Detections by SS and DeepAnomaly are presented with respectively green and red coloring. Overlapping detections by SS and DeepAnomaly become yellow.
